# SG-APSIC1175: Reusing of single-use devices management

**DOI:** 10.1017/ash.2023.108

**Published:** 2023-03-16

**Authors:** Nanthipha Sirijindadirat

**Affiliations:** President, Central Sterilizing Services Association, Bankok, Thailand

## Abstract

**Objectives:** Single-use devices are supposed to be used only once, but under some conditions those devices need to be reused. Therefore, we conducted the “Reuse of Single-Use Devices Management” project in our hospital. We evaluated single-use devices that are reused for the following factors: (1) reuse of the single-use device with the same patient; (2) a manufacturer stop production order; (3) lack of devices in inventory; and (4) device value >5,000 Thai baht (US $150). Every unit in the hospital is able to handle and monitor the reuse of single-use devices systematically for patient safety. We performed a quality surveillance project to monitor and prevent patient infection and injury from worn-out reused single-use devices, and we collected data related to the cost of reusing single-use devices. **Methods:** In a working group that studied single-use devices, responsibilities and roles were assigned and the purposes and scope of work were established. We reviewed the reuse of single-use devices policy. We created a request form for the reuse of single-use devices and a quality record form for use in units that reused single-use devices. We analyzed outcomes, monitored data, and audited the completion of these forms on these units. We assured the completion of single-use device registration. We measured the rate of reuse of single-use devices. We monitored the incidence of surgical wound infections related to reuse of a single-use device. **Results:** Both of these forms were implemented at 100%, and the number of surgical wound infections was zero. **Conclusions:** The project focused on single-use device registration and the rate of devices ready to use. The uptake of new procedures was 100%, and the expected number of surgical wound infections in patients was zero.

